# Introduction of Robotic Abdominal Wall Reconstruction Into a UK-Abdominal Wall Reconstruction Practice: Early Outcomes and Economic Analysis

**DOI:** 10.3389/jaws.2025.13710

**Published:** 2025-03-13

**Authors:** T. L. Ward, Z. Al-Amiedy, P. Robinson, A. Sharma, S. McClean, C. J. Walsh, G. S. Simpson

**Affiliations:** Wirral University Teaching Hospitals, Birkenhead, United Kingdom

**Keywords:** cost analysis, Rives Stoppa, robotic, robotic abdominal wall surgery, abdominal wall reconstruction

## Abstract

**Introduction:**

There is strong evidence that robotic abdominal wall reconstruction (AWR) reduces length of stay and postoperative complications. Despite this, it remains significantly limited in publicly funded healthcare systems due to reported costs and limited access to robotic surgical platforms.

**Methods:**

Cases were reviewed from a prospectively maintained database of AWR patients in a single unit undergoing Robotic Rives-Stoppa repair or open Rives-Stoppa repair. A prospectively maintained database was accessed and analysed. Data analysed included demographics, comorbidities, hernia characteristics, operative times and surgical outcomes. Cost analysis was performed based on length of stay, critical care bed days, and cost of consumables.

**Results:**

Data were collected from 28 robotic Rives-Stoppa repairs and 18 open Rives-Stoppa repairs. There was no difference in operative time between the two groups (199 min vs. 186 min, *p* = 0.147). The anaesthetic time was shorter in the robotic group (36 min vs. 56 min, *p* = <0.001), and the length of stay was longer in the open group (2 days vs. 7 days, *p* = <0.001). There were five critical care unit bed days in the open group, vs. 0 in the robotic group (*p* = <0.001). Complications were not significantly different (10.7% vs. 22.0%, *p* = 0.407), and there were no cases of postoperative mortality. Cost analysis showed an average saving of £1,807.58 per case.

**Conclusion:**

Our series demonstrates that robotic Rives-Stoppa AWR can be delivered in a safe manner with financial savings and equivalent operative time compared with open surgery.

## Introduction

Incisional hernias occur in 10%–20% of patients who have undergone major abdominal surgery [1]. This causes significant patient morbidity and impact on quality of life. The resultant incisional hernia surgery represents a considerable resource burden for increasingly strained healthcare systems [2]. In addition to efforts aimed at hernia prevention, it is thus important to seek robust, durable and cost-effective treatment modalities with which to treat these patients.

Abdominal wall reconstruction (AWR) has become increasingly subspecialised with an expanding variety of techniques that can be deployed to treat patients with large or complex hernias [3]. Minimally invasive approaches are gaining increasing popularity in a bid to reduce post-operative morbidity and length of stay [4]. While the initial costs of robotic surgery are higher than those of open surgery, published data suggests that robotic surgery may be financially viable when considered across the entire patient journey [5]. There is strong evidence that robotic abdominal wall reconstruction reduces the length of stay and postoperative complications such as surgical site infections [6]. Nevertheless, robotic hernia surgery remains significantly limited in publicly funded healthcare systems due to reported costs and limited access to robotic surgical platforms.

Our hospital is a UK NHS District General Hospital. We provide a dedicated AWR service employing a variety of techniques including component separation, pre-operative Botox, pre-operative pneumoperitoneum (PPP) and intra-operative fascial traction. Our unit includes a multi-disciplinary team (MDT) and subspeciality work and meetings. Here, we present data on our initial experience with robotic AWR to date and aim to demonstrate that robotic AWR can be introduced safely and economically, without compromising existing robotic surgical services. Further, we hope that this will help support the successful submission of business cases for such services in other centres in the UK and Europe.

## Methods

A prospectively maintained database of AWR patients in a single unit with a tertiary subspeciality practice in abdominal wall reconstruction was accessed and analysed. We reviewed all cases of patients undergoing ventral hernia repair via complete retro-rectus dissection and mesh reinforcement (Rives-Stoppa repair) over a 2-year period. We compared patients who underwent an open Rives-Stoppa repair (oRS) with those who underwent a robotic Rives-Stoppa repair (rRS). Patients who underwent simultaneous component separation, bowel or visceral surgery, and plastic surgery techniques (e.g., abdominoplasty) were excluded from our analysis.

Data were collected from the Electronic Patient Record (Cerner Inc., Missouri, United States) including demographics, theatre time, length of stay (LOS) on the ward, length of planned/unplanned admission to the critical care unit (CCU), American Society of Anaesthesiologists (ASA)classification, Charlson comorbidity index, primary vs. recurrent hernia repair, complications, and mortality. All patients were followed up virtually at 30 days post-operatively to assess for complications and readmissions. Complications were identified by face-to-face follow-up in the outpatient clinic, and by review of the electronic record to identify readmissions within our unit. Complications were defined as deviations from the expected postoperative course leading to a change in standard care. These were recorded using the Clavien-Dindo severity classification. Urinary retention was included as a complication in the robotic group, but not in the open group, because in the open group, a catheter was left *in situ* post-operatively as part of standard care.

Hernia defect size was measured at the maximum transverse diameter of the largest hernia defect using coronal pre-operative cross-sectional imaging, which is part of the standard pre-operative workup.

Statistical analysis was performed using Microsoft Excel and GraphPad for Windows. Continuous non-parametric data were analysed using the Mann-Whitney U test. Categorical data were analysed using Fisher’s exact test. Results with a *p*-value less than or equal to 0.05 were considered statistically significant.

### Minimally Invasive Operative Technique

Robotic-assisted AWR is performed using the DaVinci Xi^®^ robotic platform (Intuitive Surgical, Sunnydale, CA, United States). Patients undergo induction of analgesia and are placed in the supine position with arms placed at their sides and a 10-degree break in the table at the level of the iliac crest. An extended totally extraperitoneal approach (eTEP) is utilised to gain access to the ipsilateral retrorectus space. Blunt dissection using a laparoscope is conducted to provide sufficient space for the insertion of 3 mm × 8 mm robotic ports just medial to the linea semilunaris and lateral robot docking. A full dissection of the ipsilateral retrorectus space is performed. The ipsilateral posterior sheath is divided 1 cm lateral to the linea alba to gain access to the preperitoneal plane with crossover to the contralateral retro-rectus space and concurrent hernia reduction. A complete dissection of the retrorectus space is undertaken extending from the Cave of Retzius inferiorly to the subxiphoid space superiorly. The anterior sheath is closed along with the plication of any associated diastasis using a continuous, non-absorbable 0-barbed suture (V-Loc, Medtronic). Peritoneal defects are closed with an absorbable 2.0 barbed suture. Mesh reinforcement is performed using medium-weight, macroporous polypropylene mesh inserted into the retrorectus space. Local anaesthetic is infiltrated into the retrorectus plane under visualisation and drains are not routinely used. Standard postoperative analgesia is made with oral analgesics only.

### Open Operative Technique

Open abdominal wall reconstruction is performed following neuraxial blockade and induction of general anaesthesia. Patients are placed in the supine position, with their arms at their sides, and a urinary catheter is inserted. A midline laparotomy, with or without scar excision, is performed, with preservation of the hernia sac when possible. The rectus muscles are identified and an incision through the anterior sheath is performed at the medial edge of the rectus muscles. The retrorectus space is entered, with cephalad to caudad progression of dissection. Complete dissection of the retrorectus space is undertaken extending from the Cave of Retzius inferiorly to the sub-xiphoid space superiorly. Peritoneal defects are closed with a 2-0 PDS suture. Mesh reinforcement is performed using medium-weight, macroporous polypropylene mesh inserted into the retrorectus space. The medial edges of the rectus sheath are closed using the 2-0 PDS small bites technique. The skin is closed with 3-0 Monocryl and a full-length vacuum dressing is sited over the midline laparotomy wound, with 100mmHg suction, and left in place for 7 days. Post-operatively patients receive patient-controlled analgesia with intravenous opiates, which is stopped once pain is tolerated, and concurrently the urinary catheter is removed.

## Results

**FIGURE 1:**
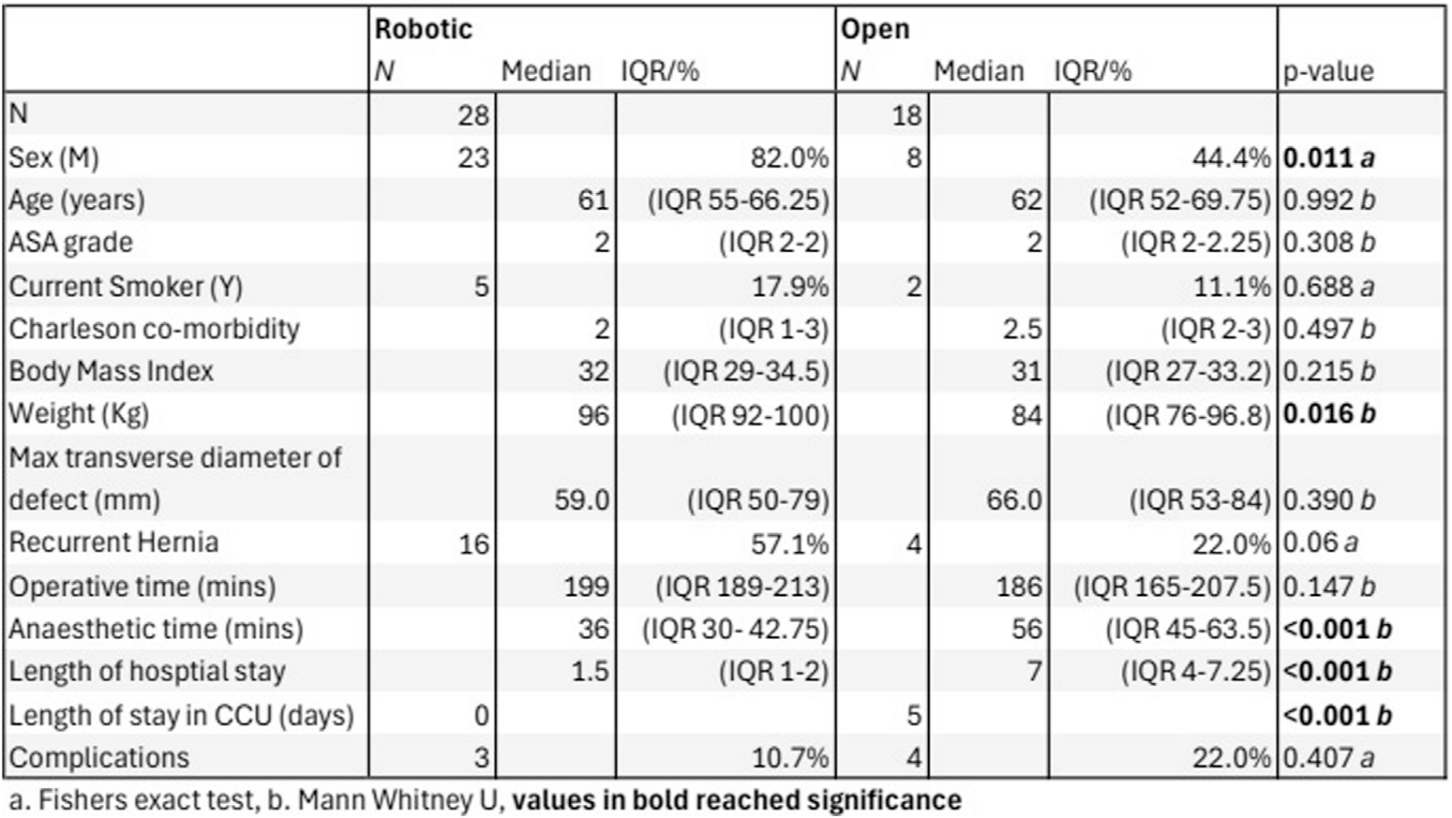
Open vs. Robotic demographics, hernia characteristics and surgical outcomes.

Between 1 April 2022 and 31 March 2024, 69 patients underwent ventral hernia repair by Rives-Stoppa reconstruction. A total of 23 patients were excluded from the analysis based on our exclusion criteria: 18 subjects underwent component separation via transversus abdominus release, 4 underwent significant intraoperative abdominoplasty by a plastic surgeon, and 1 underwent concurrent bowel resection. Of the remaining 46 patients, 28 patients underwent robotic Rives-Stoppa (rRS) and 18 underwent open Rives-Stoppa (oRS) (Figure 1). The demographic data showed that there was a significant difference in the sex distribution between the two groups, with an 82% male cohort in the rRS group vs. a 44.4% male cohort in the oRS group. There was no significant difference in the age, body mass index (BMI), ASA grade, smoking status, Charlson comorbidity index, maximum transverse diameter of the defect, or proportion of patients with recurrent hernias undergoing the procedure. The median weight of the rRS group was 96 Kg vs. 84 Kg in the oRS group, which was a statistically significant difference.

There was no significant difference in overall case length. The median operative time for the two groups was 207 min in the rRS group and 198 min in the oRS group. The median preoperative anaesthetic time was 36 min in the rRS group vs. 52 min in the oRS group.

Two patients in the open group were admitted to the CCU; one planned and one unplanned, amounting to 5 CCU bed days in total. There were no CCU admissions in the rRS group. Of the rRS group 8 cases were performed in a day case unit, with no on-site access to critical care or blood bank. All cases in the oRS group underwent surgery at a site with CCU access.

Median LOS on the ward was 1.5 days in the rRS group vs. 6.5 days in the oRS group. Half of the patients in the rRS group were discharged on postoperative day 1, while the shortest LOS in the oRS group was 3 days.

All of the patients were followed up in the outpatient clinic at 6 weeks postoperatively.

Three patients in the rRS group (10.7%) suffered post-operative complications. These were 2 cases of urinary retention requiring catheterisation and 1 return to theatre. The return to theatre was due to herniation through a peritoneal defect causing abdominal pain. A laparotomy was performed, the hernia was reduced, and the defect closed without the need for bowel resection.

Four patients in the oRS group (22%) experienced postoperative complications. There were 2 cases of wound infection both managed conservatively with antibiotics, 1 case of post-operative bleeding requiring embolisation, and 1 case of a return to theatre for evacuation of infected haematoma and removal of polypropylene mesh and replacement with bio-synthetic mesh.

### Cost Analysis

#### Cost of Stay

The cost for a patient to be admitted to a ward-based, level-1 hospital bed was calculated to be £345 per day, while a critical care bed cost was £1,881 per day. The median cost of postoperative stay for the rRS group was £517 vs. £2,242 in the oRS group. Reduced LOS and no requirement for critical care admission meant that postoperative costs were reduced in the robotic cohort. Of the 5 days in critical care for 18 patients, 3 days were for a single patient with a planned admission to the HDU postoperatively due to a poor respiratory baseline, while a further 2 days were for an unplanned admission, for a patient whose respiratory function deteriorated unexpectedly in the postoperative period. After consideration, the patient with the planned admission to critical care was excluded from the cost analysis, as the goal of this study is to compare the costs of undertaking a robotic vs. an open procedure. This patient clearly would not have been suitable for a robotic operation, as insufflation of the retrorectus space may well have worsened their already poor baseline respiratory function. Furthermore, the cost of an unplanned admission to CCU vs a planned admission is likely not equal. The 2 days in critical care were averaged across the cohort (2 days in 17 oRS patients), to allow an estimate to be included in the cost analysis. In larger cohorts, it seems plausible that a proportion of the robotic group would be admitted to the CCU, which may further reduce the cost difference we have seen.

#### Analgesia Costs

All open cases received spinal anaesthesia (in addition to general anaesthesia) and morphine Patient Controlled Analgesia (PCA) for post-operative pain relief. There were no cases in the rRS group that required aspinal or PCA. The estimated cost of spinal anaesthesia equipment is £30 per patient plus additional anaesthesia time (36 min in the rRS group vs. 52 min in the oRS group). PCA costs were estimated at £380 (€459) per patient for general surgery cases over 4.9 ± 2.2 days by Schuster et al in 2004 [7]. The current cost of this intervention may vary between different hospitals, and the 2004 figure may be an overestimate. However, Schuster’s calculation included the cost of trained staff able to manage the PCA equipment, and the authors feel it is the most accurate published cost available for the entire intervention.

#### Instrument Costs

Robotic surgery is associated with increased costs of surgical consumables compared to open surgery. The cost of the robotic instruments (Intuitive DaVinci Xi) required for the rRS group was £556 per case. A tray of open instruments is required for the open case, and a similar tray is opened for the closure of the robotic ports. Therefore, we have estimated similar costs for each group and excluded them from the analysis. All rRS and oRS cases were completed using a medium-weight, microporous, polypropylene mesh with comparable costs, and were therefore also excluded from the analysis. Significant additional costs are incurred from the purchase and maintenance of the robot and consoles, which must also be considered in centres without an existing robotic surgical service. Robotic surgery for urological, colorectal, and gynaecological malignancies is already established at our Trust, and we believe that this is common in many European hospitals of similar size; therefore, these costs were not considered in our analysis.

**FIGURE 2:**
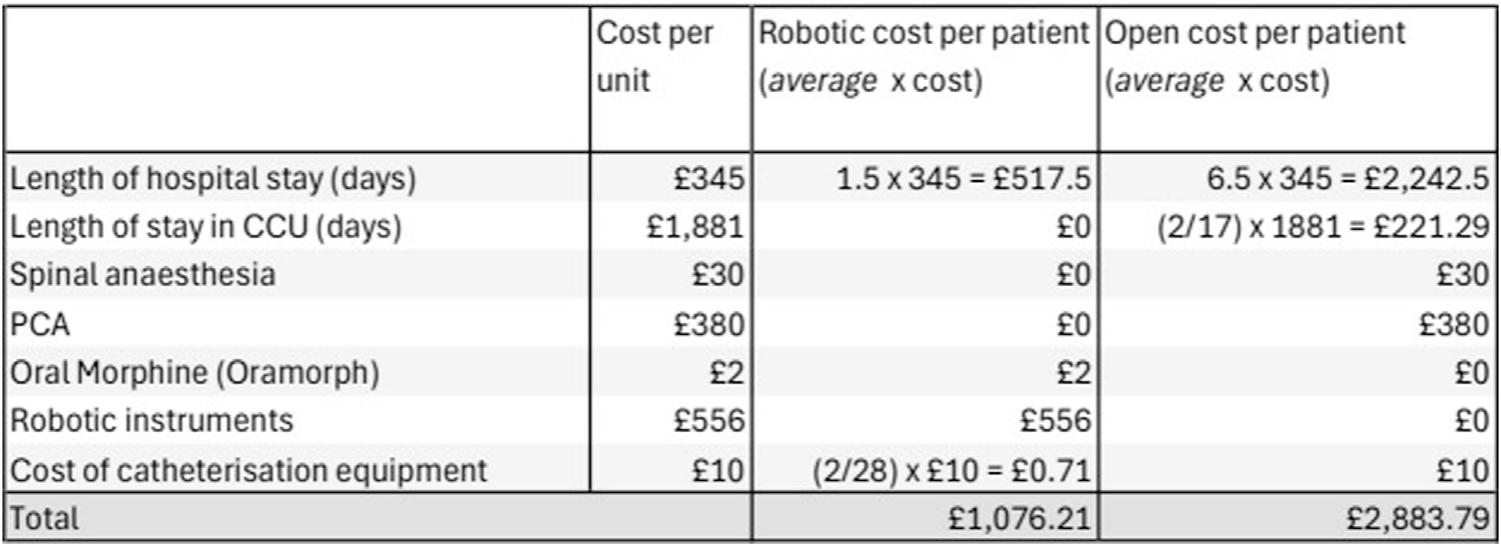
Open vs. Robotic cost-analysis based on mean outcomes.

In our series the estimated median cost-saving of rRS compared with oRS is £1,807.58 per patient based on our analysis of theatre consumables and post-operative care requirements. Within this cohort over 2 years, the introduction of robotic Rives-Stoppa hernia repair has saved an estimated £50,612.24 (Robotic cases 28 x £1,807.58) compared to the anticipated costs if all procedures were completed using the open approach. Figure 2 displays a table of the costs per case, using a mean value for the length of hospital stay and frequency of catheterisation.

## Discussion

The utilisation of robotic surgery continues to expand. Outside of the UK, robotic surgical platforms have become commonplace in the treatment of hernias. This trend has not yet been replicated in the UK, with many centres focusing their robotic provision on cancer surgery. A significant barrier to the utilisation of robotic surgical platforms in abdominal wall reconstruction is the reported increased cost of their use [8]. However our data serves to demonstrate that within a publicly funded healthcare setting, robotic AWR is not only economically feasible but clinically and financially advantageous.

Our series demonstrates that rRS can be delivered in a safe manner, with a low complication rate, providing financial benefits and equivalent operative time compared with open surgery. The introduction of robotic abdominal wall reconstruction at our centre has significantly reduced the postoperative length of stay for patients undergoing Rives-Stoppa abdominal wall reconstruction in addition to delivering an overall cost benefit. While this is not a randomised study, we have seen reduced postoperative complications in the rRS cohort, although without statistical significance, with comparable patient and hernia characteristics. We have also been able to significantly simplify our standard approach to operative intervention for AWR patients, removing the need for spinal anaesthesia, routine urinary catheterisation or patient-controlled analgesia. Consequently, we believe that robotic AWR can be implemented in a safe and cost-effective manner alongside existing robotic services.

We hope that our experience in the implementation of robotic AWR will serve to help other institutions introduce these techniques, which yield demonstrable benefits for patients and hospital trusts. We present real-world data, based in an NHS District General Hospital, and as such can be used to inform the submission of business cases in NHS Hospitals for robotic-assisted abdominal wall reconstruction. Indeed, many patients in this cohort underwent robotic Rives-Stoppa on a day theatre list, alongside a robotic anterior resection for colorectal cancer. This has had the effect of optimising our utilisation of robotic resources and theatre utilisation without compromising robotic cancer surgery.

Although we present promising initial results regarding the safe and economic integration of robotic AWR, we acknowledge that this series remains relatively small and presents only short-term, observational results. The significant sex discrepancy in the two cohorts is likely related to the small sample size, although selection bias remains possible. The weight difference of the two groups may reflect selection bias from the operating surgeon, as they may conscider the risk of oRS greater than rRS, particularly in patients with a higher BMI. This has been demonstrated in other AWR cohorts comparing open vs. robotic intervention [9]. Furthermore, the weight discrepancy may well be related to the sex discrepancy in the two groups, with the rRS group having a greater number of men and a higher average weight. As a retrospective review of a cohort, there was no allocation to the open or robotic groups. Patients may have been deemed unsuitable for a robotic operation if they had a particularly large hernia defect, or multiple previous operations that left a significantly scarred abdomen. This was indicated in our data with the average defect size being larger in the open group, although it did not reach statistical significance. This may be further confounded by the high number of women and lower average weight in the open group, who due to smaller stature, may have a proportionally larger hernia defect. Further work is ongoing to evaluate longer-term benefits, including hernia-related quality of life.

We acknowledge that our cost analysis is limited to theatre consumables, operative time and postoperative care and does not consider costs associated with setting up a robotic service as robotic cancer surgery was already established within the Trust.

Despite the potential weaknesses of our article, listed above, this paper demonstrates that even in the very early adoption phase associated with an early learning curve, robotic AWR can be safely implemented and yield a financial benefit from its inception. We also believe that these benefits will continue to improve as our experience with robotic AWR continues to grow.

The authors believe that robotic-assisted AWR is now a key component of our AWR service and takes its place as a technique that complements our existing range of AWR techniques, including open Rives-Stoppa and component separation, pre-operative abdominal wall botulinum toxin injection, pre-operative pneumoperitoneum and intra-operative fascial traction. The key to the integration of this new technique lies in its appropriate utilisation based on the clinical scenario, patient factors and abdominal wall/hernia anatomy which should be guided by a specialist AWR MDT. We believe that our success in developing our robotic AWR service has been built on a strong foundation of significant experience in open AWR surgery, and it is vital to establish this basis before implementing robotic AWR. With further experience we have expanded our robotic repertoire of AWR procedures to include Transversus Abdominis Release (TAR) and complex parastomal hernia repair. This has been achieved by building on our strong foundation of experience in rRS.

## Data Availability

The raw data supporting the conclusions of this article will be made available by the authors, without undue reservation.
